# The impact of supply chain management on a company’s operation and decision based on the multidimensional data analysis of upstream and downstream industry market states

**DOI:** 10.7717/peerj-cs.1369

**Published:** 2023-06-13

**Authors:** Jingwen Ding

**Affiliations:** Faculty of Business, Monash University, Melbourne, Victoria, Australia

**Keywords:** Supply chain management, Market status of upstream and downstream industries, Multimode joint data analysis, LMBP feedback neural network, Company operation decision

## Abstract

This study provides data analysis support for the entire enterprise procurement management process, thereby improving the management effectiveness of supply chain operations. It analyzes upstream and downstream industry market status data in the supply chain and various primary data in enterprise management activities. By utilizing the Delphi method to screen and verify multimode market status data indicators, which significantly impact upstream and downstream industries in multiple rounds, 28 types of market status data were selected for analysis. This analysis aimed to investigate the effect of supply chain management on operational decisions within the company. The data reduction method based on adaptive statistics was the most effective in revealing the market status and promoting efficient operation decision-making based on supply chain management. This study also suggests a brand-new technique for measuring supply chain performance based on the Levenberg-Marquardt Back Propagation (LMBP) algorithm, offering a more impartial manner of doing so. The performance evaluation results showed a maximum error level of less than 0.4% when paired with empirical analysis. The proposed optimization model provides strategic guidance for optimizing supply chain management and improving overall performance.

## Introduction

The competition in the 21st century has changed from the match between enterprises to the competition between supply chains (Deng et al., 2020). As the transformation progresses, the company will use multidimensional data analysis to assess the state of the upstream and downstream industrial markets to manage the supply chain more efficiently. This will effectively aid the business in operational decisions and ensure the implementation of cost-cutting and efficiency-boosting measures (Zhang et al., 2021).

More businesses are beginning to value the impact of multidimensional data analysis on the market status of upstream and downstream industries, use data analysis techniques to direct production and operations, and accomplish scientific decision-making as a result of the wave of data analysis (Rudskoi & Baurova, 2019). The research shows that the analysis based on the multidimensional data analysis of upstream and downstream industry market status plays an essential role in business understanding, performance measurement, improving customer relations, and creating business opportunities (Li & Li, 2016). For example, the management knowledge mined through data analysis can help enterprises identify the key factors that affect product quality in the supply chain, summarize the causal relationship between product quality and production processes, and thus avoid potential risks (Qu, Liu & Bao, 2021). It can also help enterprises understand customer needs, strengthen customer loyalty, maintain a good relationship with customers, and solve problems before crises (Mishra et al., 2018). In brief, adept utilization of practical operational data analysis and management knowledge mining can facilitate the acquisition of actionable, evidence-based insights and knowledge from internal and external operational data sources. This, in turn, empowers enterprises to make prompt and informed decisions, allowing managers to understand their organization’s standing better, consequently enhancing work efficiency and uncovering novel business prospects (Bian, Shang & Zhang, 2016).

Many scholars have studied the positive impact of multimodal joint data analysis on promoting the company’s operational decisions. Fan, Lau & Zhao (2015) empirically discussed the role of data analysis capability in group strategy, culture, and financial control by establishing a structural equation model. The study’s findings indicate that data analysis resource integration capabilities most influence group strategy, culture, and financial control. Kiseľáková et al. (2018) highlighted the moderating impact of enterprise strategic orientation while examining the relationship between data analysis and enterprise performance; specifically, businesses should build a data-driven strategy from a macro viewpoint to receive twice the outcome with half the effort. When exploring the relationship between data analysis and enterprise performance, the intermediary role of dynamic capabilities was emphasized (Perfilieva et al., 2016). Making decisions about how a company operates is a key contributor to its performance and a crucial safeguard for its competitiveness. Wang, Wang & Huang (2017) pointed out in his research that the reduction of costs and the improvement of customer satisfaction are primarily due to enterprises’ efficient operation decision-making ability. Kościelniak & Puto (2015) has also concluded that operational decision-making can promote an enterprise’s customer service capabilities, increase customer service satisfaction, and thus improve other performance. Flynn, Koufteros & Lu (2016)’s research confirmed the conclusion that the external integration of the supply chain (customer integration and supplier integration) positively affects performance. Taking Chinese manufacturing enterprises as the survey object confirmed the intermediary role of supply chain integration in relationship and operation performance. Many scholars focus on different aspects of evaluating the performance of enterprise operations and decision-making. Cost, flexibility, service, quality, and other indicators are used to analyze and assess the enterprise operation performance. For example, Centobelli, Cerchione & Esposito (2018) used service level, delivery capacity, and financial status to evaluate the performance of enterprise operation decisions. Truong et al. (2017) categorized operational performance into cost, flexibility, and distribution for evaluation. Belekoukias, Garza-Reyes & Kumar (2014) argues that operational performance is manifested through the rapid response ability, production cost, delivery cycle, and customer satisfaction of enterprises. From this perspective, Belekoukias, Garza-Reyes & Kumar (2014) discusses the key indicators that enable enterprises to gain a competitive edge in the market. Rompho (2018) evaluated enterprise operational decision-making performance using four distinct criteria: new products, services, quality, and cost.

Most scholars have recognized the positive role of data analysis capability in improving enterprise performance, and data analysis is also considered an essential driver of innovation (Nasution, Fahmi & Prayogi, 2020). However, most of these studies focus on direct effects, and there are few kinds of literature discussing the role of other factors in the promotion path, let alone empirical research. The interpretive structure model is used in this study to decompose ten hierarchical relationships among 28 different types of market data to aid cross-border e-commerce decision-makers in understanding the rational relationships among indicators and building a valuable and efficient supply chain system. The LMBP feedback neural network model is used to automatically update the weight of each indicator and evaluate cross-border e-commerce enterprises.

## Related Research Works

Because more competitors and clients have a more comprehensive range of needs in the upstream and downstream industrial marketplaces, businesses are forced to make more operational decisions due to the growing product diversity (Wu et al., 2021). To inform their operations and decision-making, businesses must employ scientific methods to study the upstream and downstream industrial markets and judiciously integrate multidimensional data analysis of the upstream and downstream industrial market status (Gardi et al., 2021). Nhemachena & Murimbika (2018) confirmed that data analysis capability could positively affect enterprise performance by improving supply chain management capability and company reputation. Chowdhury & Quaddus (2017) improved the value of the supply chain through data analysis, researched the main influencing factors of the multimodal joint data of market status, and proposed the dynamic capability theory. Kaur et al. (2020) established a procurement model based on the supply chain to promote data analysis on the whole supply chain process. Wang et al. (2018) analyzed the crisis and opportunities faced by traditional supply chain cost control methods in the e-commerce industry. By utilizing the ease of information sharing, Craighead, Ketchen Jr & Darby (2020) emphasized that businesses should incorporate the supply chain management theory into their cost management practices based on the multidimensional data analysis of the upstream and downstream industry market status. This will improve the overall level of cost control for businesses. Reda et al. (2020) discussed using supply chain data analysis to make up for traditional cost management methods and studied new supply chain cost control strategies and enterprise product pricing models. Schmidt & Wagner (2019) studied supply chain management for analysis and research and analyzed the functional role, scenario mode, and performance evaluation of supply chain management. Lima-Junior & Carpinetti (2020) positioned the relationship between enterprises and developed a new supply chain performance evaluation method. The author graded the supply chain management and proposed that the lead time of goods and the service level of retailers are crucial to the optimal decision-making and pricing of enterprise operations.

Data analysis based on supply chain management is a descriptive, predictive, and normative analysis of multimodal joint data based on upstream and downstream industries’ market status and operation data (Tiwari, Wee & Daryanto, 2018). In addition to statistical analysis, multimodal joint data analysis can combine data collection, storage, knowledge management, and analysis to extract useful information from many upstream and downstream industry market status data, providing a reference for decision-making (Tavasszy, 2018). In order for decision-makers to understand the changing trend of the enterprise’s external environment and to facilitate their analysis, Ouedraogo, Montarnal & Gourc (2022) believes that data analysis based on supply chain management can efficiently sort out upstream and downstream enterprise data as well as user-generated content. Goodarzian et al. (2021) proposed a demand framework for the supply chain of the medical industry based on the data analysis of upstream and downstream enterprises in the medical industry, which can effectively improve the efficiency of operational decision-making. The framework has been proven to be effective by experts in related fields. Sang (2021) introduced the rough set and BP neural network into the supply chain performance evaluation model and set the target error level within 10%. After reducing the index system in the balanced scorecard, they brought them into the neural network for sample training. The case proved that the performance evaluation value was consistent with the result. The mean square error level meets the set value. Yan et al. (2019) used the frequency analysis method and analytic hierarchy process to screen out evaluation indicators and obtained reasonable and effective evaluation results through sample training and analysis. On this basis, they continued to optimize supply chain performance and developed practical suggestions for optimizing enterprise supply chain performance. Based on the aforementioned context, this study develops a data reduction technique based on adaptive statistics, develops a supply chain evaluation index system based on a review of relevant theories of supply chain performance evaluation, and employs an LMBP feedback neural network model to assess the index system to support the efficient operation of the enterprise supply chain.

## Multidimensional Data Analysis on the Market Status of Upstream and Downstream Industries

### Multimode market status data selection

The manufacturing sector includes a wide variety of businesses. The completely different product types and manufacturing process characteristics of various manufacturing enterprises in the upstream and downstream sectors (such as electronic component manufacturing enterprises, small household appliance manufacturing enterprises, and food manufacturing enterprises) will result in the production of large amounts of multimodal joint data (Wang et al., 2022). In recent years, China’s auto industry has increased its production capacity, resulting in a huge increase in exports. Cross-border e-commerce is becoming a new track for Chinese auto brands to go overseas, helping Chinese manufacturers seize more overseas opportunities. China’s flat replacement auto parts have become the global auto parts cost-effective supermarket; with the development of autonomous driving, the Internet of Vehicles and other technologies, the demand for Chinese-made auto supplies have surged in Europe and the United States. This study selects automobile manufacturing enterprises in China’s listed manufacturing industry as research samples. The Delphi method (Belton et al., 2019) is used with industry characteristics to screen and validate upstream and downstream industries’ multimode market status data indicators with a significant influence. Twenty-eight types of market status data are selected for analysis to study the impact of supply chain management on the company’s operational decisions. The multimode market status data of upstream and downstream industries are shown in Table 1.

**Table 1:** Supply chain multimode joint data.

**Num.**	**Data**	**Num.**	**Data**
a1	Transportation and arrival data of raw materials	a15	Demand forecast data
a2	Process operation data of raw materials	a16	Transportation process monitoring data
a3	Source of product components and production process data	a17	Order Scheduling and Production Execution Data
a4	Product quality inspection data	a18	Supply source data
a5	Supply and consumption data of components	a19	Supplier production progress data
a6	Scrap and return quantity and quality data	a20	Supplier Delivery Data
a7	Working status data of production equipment	a21	Customer order data
a8	Production equipment maintenance data	a22	Channel real-time sales data
a9	Warehousing environment awareness data	a23	Customer personalized demand data
a10	Processing environment perception data	a24	Social event data
a11	Various plan data	a25	Short-term trend data
a12	Various inventory data	a26	Long-term trend data
a13	Production control data	a27	Supply Chain Risk Data
a14	Complete set of material data	a28	Political, economic, and weather data

The massive multidimensional data analysis of upstream and downstream industry market status can significantly improve the efficiency of enterprise operation decision-making and promote enterprises to gain more in-depth insight into the supply chain through multi-angle and multi-level data analysis. However, it is unwise to analyze big data directly in the age of big data. Not every kind of data can play a positive role for extensive data, and redundant and noisy data often play a negative role. Therefore, it is necessary to reduce the volume of data through data reduction technology to reduce the computational complexity and improve the efficiency of analysis and mining.

### Data reduction method based on adaptive statistics

The extraction of key factors of supply chain performance is a process of dimensionality reduction and feature selection of high-dimensional data. Principal components analysis (PCA) is a method to simplify data sets. Using dimension-reduction ideas is a commonly used method to transform multiple attributes into a few extended attributes. However, the traditional PCA algorithm is based on the sample correlation coefficient or covariance matrix. The research of robust statistics shows that these two statistics lack tolerance for outliers. Therefore, the traditional PCA algorithm is significantly affected by outliers and unsuitable for direct analysis of performance data of complex supply chains. In addition, traditional PCA only examines the linear relationship between variables, which restricts its use in practical analysis. To improve the performance of traditional PCA, this study proposes an enterprise operational data reduction method based on Adaptive statistics. It selects statistics with good robustness and easy calculation as indicators. The robust estimation uses the following common statistics as scale parameters, as shown in Eqs. (1)–(5).

Mean absolute deviation.

(1) \begin{eqnarray*}\mathrm{AD}= \frac{1}{\mathrm{N}} \sum _{\mathrm{k}=1}^{\mathrm{n}}{|}{\mathrm{x}}_{\mathrm{ k}}-\text{median}({\mathrm{x}}_{\mathrm{k}}){|}.\end{eqnarray*}

Median absolute deviation

(2) \begin{eqnarray*}\mathrm{MAD}=1.482\text{median} \left\{ \left\vert {\mathrm{x}}_{\mathrm{k}}-\text{median} \left( {\mathrm{x}}_{\mathrm{k}} \right) \right\vert \right\} .\end{eqnarray*}

Quartered layout

(3) \begin{eqnarray*}{\mathrm{d}}_{\mathrm{F}}={\mathrm{F}}_{\mathrm{U}}-{\mathrm{F}}_{\mathrm{L}}\end{eqnarray*}

Q_n_ scale:

(4) \begin{eqnarray*}{\mathrm{Q}}_{\mathrm{n}}& =2.22\mathrm{ \ast }1.19 \left\{ \left\vert {\mathrm{x}}_{\mathrm{i}}-{\mathrm{x}}_{\mathrm{j}} \right\vert ;\mathrm{i}\lt \mathrm{j} \right\} \end{eqnarray*}

(5) \begin{eqnarray*}\mathrm{s}& = \left( \begin{array}{@{}c@{}} \displaystyle \mathrm{h}\\ \displaystyle 2 \end{array} \right) \approx \left( \begin{array}{@{}c@{}} \displaystyle \mathrm{n}\\ \displaystyle 2 \end{array} \right) ,\mathrm{h}= \left[ \frac{\mathrm{N}}{2} \right] +1.\end{eqnarray*}

In this study, an adaptive robust scaling parameter estimation method is proposed, and its iterative algorithm is as follows:

Step 1. Initialize the number of iterations i = 0, and calculate the initial estimator of the scale parameter ${\hat {\sigma }}_{0}$.

Step 2. Sample standardization: ${\hat {\mathrm{x}}}_{\mathrm{k}}= \frac{{\mathrm{x}}_{\mathrm{k}}}{{\hat {\sigma }}_{\mathrm{i}}} ,\mathrm{k}=1,2,\ldots ,\mathrm{N}$.

Step 3. Estimate the scale score function: take g(⋅) as the basis function, and take the polynomial function: ${\mathrm{g}}_{\mathrm{j}} \left( \mathrm{u} \right) ={\mathrm{u}}^{\mathrm{j}}$.

The scale score function is $\varnothing \left( \mathrm{x} \right) ={\hat {\mathrm{a}}}^{\mathrm{T}}\ast \mathrm{g}(\mathrm{x})$, Where the calculation of $\hat {\mathrm{a}}$ are shown in Eq. (6)

(6) \begin{eqnarray*}\hat {\mathrm{a}}={ \left( \sum _{\mathrm{k}=1}^{\mathrm{N}}\mathrm{g}({\hat {\mathrm{x}}}_{\mathrm{ k}}){\mathrm{g}}^{\mathrm{T}}({\hat {\mathrm{x}}}_{\mathrm{ k}}) \right) }^{-1}\sum _{\mathrm{k}=1}^{\mathrm{N}}{\hat {\mathrm{x}}}_{\mathrm{ k}}\mathrm{g}({\hat {\mathrm{x}}}_{\mathrm{k}}).\end{eqnarray*}

Step 4. Solve ${\mathop{\sum }\nolimits }_{\mathrm{k}=1}^{\mathrm{N}}\varphi ( \frac{{\mathrm{x}}_{\mathrm{k}}}{\sigma } )=0$, Update scale parameter estimates ${\hat {\sigma }}_{\mathrm{i}}\rightarrow {\hat {\sigma }}_{\mathrm{i}+1}$.

Step 5. Test convergence: Terminate if ${|}{\hat {\sigma }}_{\mathrm{i}+1}-{\hat {\sigma }}_{\mathrm{i}}{|}\lt {|}{\hat {\sigma }}_{\mathrm{i}}{|}$, Otherwise, i → i + 1, return to Step 2.

We compared the robustness of these types of statistics through numerical experiments to screen out projection indicators with better robustness. To investigate the robustness of statistics, we randomly generate a certain number of samples according to a specific distribution and then create a group of samples with another distribution type as the pollution distribution. By adjusting the ratio of original samples to noise, the pollution rate *ɛ*, examine the changes in statistics to determine whether the object under investigation has a certain tolerance.

Epsilon We set $\mathrm{X}{\mathrm{C}}_{1}\sim \left( 1- \right) \mathrm{N} \left( 0,1 \right) +{\chi }^{2} \left( 2 \right) ,\mathrm{X}{\mathrm{C}}_{2}\sim \mathrm{N} \left( 0,1 \right) +\text{Weibull}(1,2).$ represent the contamination rate vector of the sample. The ith statistic in the pollution rate *ɛ*- The sequence formed under j is *σ*- The change rate of defined scale parameters is shown in Eq. (6):

We set $\mathrm{X}{\mathrm{C}}_{1}\sim \left( 1- \right) \mathrm{N} \left( 0,1 \right) +{\chi }^{2} \left( 2 \right) ,\mathrm{X}{\mathrm{C}}_{2}\sim \mathrm{N} \left( 0,1 \right) +\text{Weibull}(1,2).$ represents the contamination rate vector of the sample. The ith statistic in the pollution rate *ɛ*_j_ The sequence formed below is *σ*^i^(XC_j_). The change rate of defined scale parameters is shown in Eq. (7):

(7) \begin{eqnarray*}{\delta }_{\mathrm{j}}^{\mathrm{i}}= \frac{{|}{\sigma }^{\mathrm{i}} \left( \mathrm{X}{\mathrm{C}}_{\mathrm{ j}} \right) -{\sigma }^{\mathrm{i}} \left( \mathrm{X} \right) {|}}{{\sigma }^{\mathrm{i}} \left( \mathrm{X} \right) } .\end{eqnarray*}

**Figure 1:**
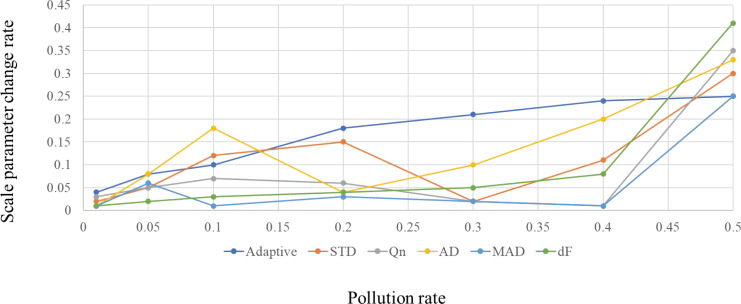
Comparison of the robustness of the scale parameters based on the chi-square distribution.

**Figure 2:**
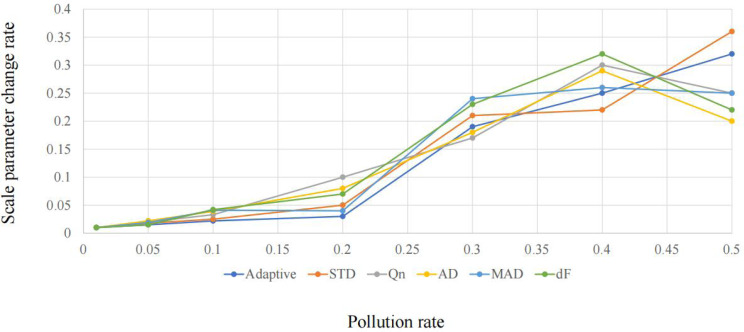
Comparison of the robustness of scale parameters based on Weibull distribution.

The relationship between scale parameter change rate and pollution rate is shown in Figs. 1 and 2.

It can be seen from Figs. 1 and 2 that adaptive statistics have good robustness in the case of high noise. Therefore, this study uses adaptive statistics as the projection index function to characterize the dispersion of high-dimensional data on a one-dimensional projection and obtain a robust data reduction and dimension reduction method.

**Figure 3:**
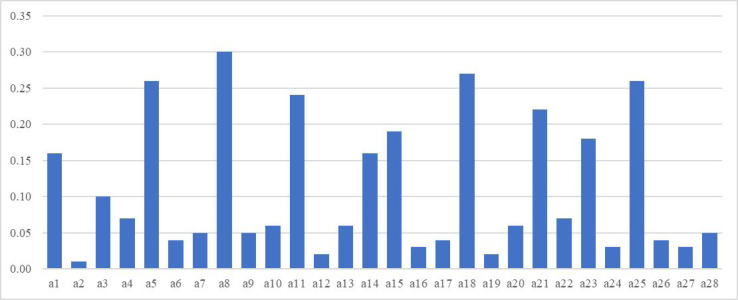
Calculation of the criticality of the Wine dataset based on RPP-PCA.

We identified a feature subset with high criticality among 28 types of market status data using the data reduction technique of adaptive statistics, which can best reveal the market state and support the effectiveness of business operation decision-making based on supply chain management. Figure 3 shows the calculation results of the criticality of 28 types of data indicators. Variables 1, 5, 8, 11, 14, 15, 18, 21, 23 and 25 are significant. Therefore, the feature subset we set up is X_1_, X_2_, X_3_, X_4_, X_5_, X_6_, X_7_, X_8_, X_9_, X_10_, which includes the above 10 data indicators, including raw material transportation and arrival data, component supply and consumption data, production equipment working status data, various planning data, material integrity data, demand forecast data, supply source data, customer order data, customer personalized demand data and short-term trend data. It represents the critical data representing the market status of upstream and downstream industries.

## A Company Operation Decision Model Based on Supply Chain Management Data

To build a model suitable for evaluating cross-border e-commerce supply chain performance, this study designed the LMBP algorithm by combining the BP and LM algorithms. While maintaining the convergence characteristics of the gradient steepest descent method, it also has the convergence speed of the Gauss-Newton method. N represents the number of input nodes, M represents the number of output nodes, jth represents the hidden layer unit, i ^th^ represents the input layer unit, net_pj_ represents the input value to the hidden layer, x_pj_ represents the value of the input layer unit, w_ji_ represents the weight, w_jo_ represents the threshold, g(net_pj_) represents the excitation function of the hidden layer, w_ko_ is the threshold of layer k, y represents the target output, $\hat {\mathrm{y}}$ represents the network output, ${\hat {\mathrm{y}}}_{\mathrm{pk}}$ is the target output of the k^th^, and *η* is the learning rate under the Newton method.

The sum of the mean square error of the LMBP network is

(8) \begin{eqnarray*}\mathrm{E}= \frac{1}{2} \sum _{\mathrm{p}=1}^{\mathrm{N}}\sum _{\mathrm{k}=1}^{\mathrm{M}}({\mathrm{y}}_{\mathrm{ pk}}-{\hat {\mathrm{y}}}_{\mathrm{pk}}).\end{eqnarray*}

The input value of the hidden layer and the excitation function of the hidden layer are, respectively:

(9) \begin{eqnarray*}{\mathrm{net}}_{\mathrm{pj}}& =\sum _{\mathrm{j}=0}^{\mathrm{N}}{\mathrm{w}}_{\mathrm{ jt}}{\mathrm{x}}_{\mathrm{pi}}\end{eqnarray*}

(10) \begin{eqnarray*}\mathrm{g} \left( {\mathrm{net}}_{\mathrm{pj}} \right) & =1/(1+{\mathrm{e}}^{-{\mathrm{net}}_{\mathrm{pj}}}).\end{eqnarray*}

The target output of ${\hat {\mathrm{y}}}_{\mathrm{pk}}$ is expressed by Eq. (11):

(11) \begin{eqnarray*}{\mathrm{net}}_{\mathrm{pk}}=\sum _{\mathrm{j}=0}^{\mathrm{N}}{\mathrm{w}}_{\mathrm{ kj}}\mathrm{g} \left( {\mathrm{net}}_{\mathrm{pj}} \right) +{\mathrm{w}}_{\mathrm{k}0},{\hat {\mathrm{y}}}_{\mathrm{pk}}=\mathrm{g} \left( {\mathrm{net}}_{\mathrm{pk}} \right) .\end{eqnarray*}

The weight update Eq. is:

(12) \begin{eqnarray*}\Delta {\mathrm{W}}_{\mathrm{kj}}=-{ \left[ \mathrm{H}+\mu \mathrm{I} \right] }^{-1}{\mathrm{J}}^{\mathrm{T}}\mathrm{e}.\end{eqnarray*}

**Figure 4:**
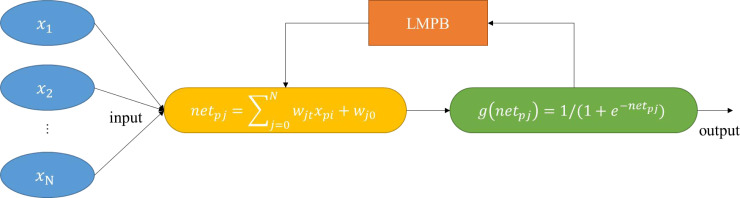
LMBP neural network structure.

Here, the I is the unit matrix, and the weight value is updated after batch training, which makes the neural network learning process more stable and rapid and can adapt to a large amount of calculation. In the modeling process, the more critical the data, the more accurate the modeling. The iteration process mainly includes four steps:

(1) Take all inputs, initialize the weight value, and variable µ(generally µ=0.01).

(2) Calculate the actual output, mean square error sum function, and Jacobian matrix of the input value.

(3) Gauss-Newton method is used to update the weights to form new parameters.

(4) Calculate the mean square error E(w) repeatedly. If the mean square error decreases, update it. If the mean square error does not drop, do not update and skip to step 3. The LMBP calculation process is shown in Fig. 4.

**Figure 5:**
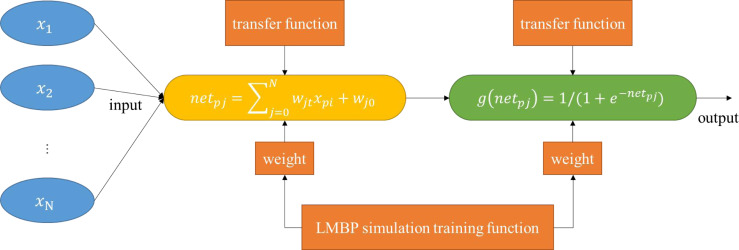
Performance evaluation model of cross-border e-commerce supply chain based on LMBP.

Supply chain management adopts the coordinated management mechanism, links and coordinates the functional organizations, realizes the transformation of internal functional management to process management, puts the enterprise itself in the chain process, and realizes the coordinated linkage of the front and back end of the organization. In this study, LMBP feedback neural network algorithm is used to build a performance evaluation model of a cross-border e-commerce supply chain based on the LMBP algorithm, as shown in Fig. 5.

**Figure 6:**
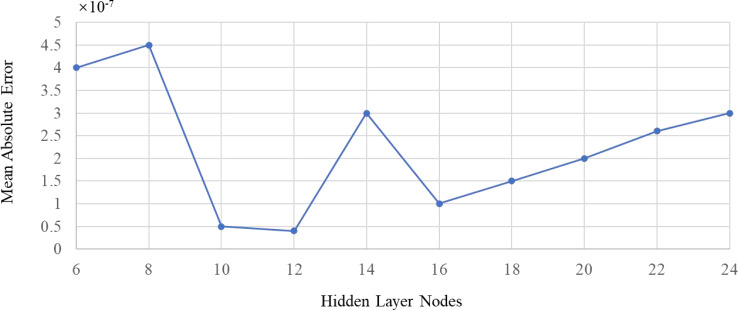
Relationship between the number of hidden layer nodes and mean square error.

The model is mainly divided into three stages to evaluate the performance of the cross-border e-commerce supply chain:

(1) Collect and process data: at this stage, it is necessary to collect data, complete the sorting and calculation of 28 evaluation indicators of the six-dimensional balanced scorecard, how complete the processing of qualitative data, how to adjust variables according to historical data, and how to normalize the actual data.

(2) LMBP algorithm is used for data training and learning: as shown in Fig. 6, the number of hidden layers, nodes, and transfer functions are selected scientifically, which makes data prediction and indicator evaluation more accurate and efficient.

(3) Training result analysis and performance optimization plan; finally, we need to analyze and process the training results in the early stage. On the one hand, we need to record the process and status of weight updating and neural network establishment. On the other hand, we need to analyze the path and scheme to improve the performance level by comparing the poor performance data with the excellent performance data.

In this study, two layers of the neural network are selected: one layer of the hidden layer and one layer of the output layer. For the number of remote layer nodes and neurons, it is necessary to ensure that there are enough and avoid excessive numbers. A disproportionate number of nodes will also lead to excessive computation and overfitting problems. Refer to the empirical Eq. (13) for the number of hidden layer nodes and the Ockham razor principle—the number of hidden layer nodes in a neural network should be less than the number of training set samples. This study analyzed 28 market status data of automobile manufacturers in China’s listed manufacturing industry in 2022. The evaluation results are 4-dimensional data; the number of nodes in the input layer R is 28, and the number of nodes in the output layer S is 4. The calculation shows that the number of hidden layer nodes is reasonable between 7–16.

(13) \begin{eqnarray*}\mathrm{n}=(\mathrm{R}+\mathrm{S})^{1/2}+\mathrm{a},1\leq \mathrm{a}\leq 10.\end{eqnarray*}

To determine the optimal number of hidden layer nodes, we used the mean square error of training prediction results as a reference to train the 12-month data in 2021, continuously increase the number of hidden layer nodes, and repeat the same data. It can be concluded from Fig. 6 that the optimal number of remote layer nodes is 12, and the MSE at this time is the minimum, which is 2.62e−12.

The traditional BP algorithm uses the gradient steepest descent to update the weight value. Although it has good convergence, it has slowed local convergence defects. The LMBP algorithm combines the Gauss-Newton method and the steepest descent method, which can maintain the convergence characteristics of the gradient-most vertical descent method and effectively improve the convergence speed of the Gauss-Newton method. By understanding the LMBP algorithm’s characteristics, this research builds a company operation model based on the LMBP algorithm. This model is combined with an evaluation index system composed of multimodal joint data of upstream and downstream industry market status. This can make establishing index weights more efficient and provide a more objective and novel method for evaluating supply chain performance.

## An Empirical Analysis of the Optimization of Corporate Operation Decision

The feature subset of the multidimensional data analysis of upstream and downstream industrial market state with high criticality is collected for this study using the adaptive statistics-based data reduction methodology. The weight of the feature subset is obtained based on the LMBP feedback neural network algorithm. Then, according to the supply chain performance analysis model and indicator system, we provide the basis for the company’s operational decisions.

Because the original data has different dimensions, it needs to be expected. Different input variables usually fall into different ranges, leading to significant variables suppressing the performance of other variables, thus affecting the ability of the model to discover laws through input and output values. Therefore, we first normalize the data to eliminate the impact of dimensions. Use Eq. (14) for processing.

(14) \begin{eqnarray*}{\mathrm{y}}_{\mathrm{i}}=({\mathrm{x}}_{\mathrm{max}}-{\mathrm{x}}_{\mathrm{i}})/({\mathrm{x}}_{\mathrm{max}}-{\mathrm{x}}_{\mathrm{min}}).\end{eqnarray*}

x_i_ represents the value of index data before normalization, y_i_ is the value after normalization, x_max_ and x_min_ represent the maximum and minimum values of the indicator data value range.

To intuitively represent the level of key data of upstream and downstream industries’ market status, we define different score ranges and divide them into four performance levels: poor, reasonable, sound, and excellent. Concerning the literature and the opinions of managers of automobile manufacturing enterprises, we obtain the performance score range shown in Table 2. At the same time, in the analysis process, the four-dimensional data of (1000), (0100), (0010), and (0001) represent poor, medium, sound, and excellent, respectively.

**Table 2:** Supply chain performance score level of S company.

**Score range of indicator performance level**	**Level**	**Output representation**
[0,0.25)	poor	(1 0 0 0)
[0.25,0.5)	reasonable	(0 1 0 0)
[0.5,0.75)	good	(0 0 1 0)
[0.75,1)	excellent	(0 0 0 1)

The performance level of each 12 months in 2021 is shown in Table 3, which is represented by data and expressed as a matrix.

\begin{eqnarray*}{T}_{0}= \left[ \begin{array}{@{}cccccccccccc@{}} \displaystyle 0&\displaystyle 1&\displaystyle 1&\displaystyle 0&\displaystyle 0&\displaystyle 0&\displaystyle 0&\displaystyle 0&\displaystyle 0&\displaystyle 0&\displaystyle 0&\displaystyle 0\\ \displaystyle 1&\displaystyle 0&\displaystyle 0&\displaystyle 0&\displaystyle 1&\displaystyle 1&\displaystyle 0&\displaystyle 0&\displaystyle 0&\displaystyle 0&\displaystyle 0&\displaystyle 0\\ \displaystyle 0&\displaystyle 0&\displaystyle 0&\displaystyle 1&\displaystyle 0&\displaystyle 0&\displaystyle 1&\displaystyle 1&\displaystyle 1&\displaystyle 1&\displaystyle 0&\displaystyle 0\\ \displaystyle 0&\displaystyle 0&\displaystyle 0&\displaystyle 0&\displaystyle 0&\displaystyle 0&\displaystyle 0&\displaystyle 0&\displaystyle 0&\displaystyle 0&\displaystyle 1&\displaystyle 1 \end{array} \right] \end{eqnarray*}

**Table 3:** Original data of supply chain performance of S company in 12 Months.

**Month**	**X1**	**X2**	**X3**	**X4**	**X5**	**X6**	**X7**	**X8**	**X9**	**X10**
January	1.00	0.96	1.00	0.84	0.47	0.51	0.71	0.99	1.00	1.00
February	1.00	0.54	1.00	0.77	0.49	0.66	0.76	0.98	1.00	1.00
March	0.75	0.51	1.00	0.72	0.50	0.52	0.82	0.99	1.00	1.00
April	1.00	0.48	1.00	0.70	0.47	0.69	0.74	0.97	1.00	1.00
May	1.00	0.47	1.00	0.64	0.46	0.64	0.68	0.98	1.00	1.00
June	0.75	0.42	1.00	0.60	0.43	0.59	0.63	0.99	1.00	1.00
July	0.75	0.42	1.00	0.71	0.41	0.55	0.62	1.00	1.00	1.00
August	0.75	0.40	1.00	0.76	0.46	0.62	0.61	1.00	1.00	1.00
September	1.00	0.49	1.00	0.74	0.47	0.68	0.78	1.00	1.00	1.00
October	1.00	0.37	1.00	0.62	0.56	0.59	0.75	0.99	1.00	1.00
November	0.75	0.44	1.00	0.83	0.51	0.63	0.94	0.98	1.00	1.00
December	1.00	0.80	1.00	0.85	0.51	0.60	0.91	0.97	1.00	1.00

The number of hidden layers in the network of the company’s operation decision-making model based on the LMBP algorithm proposed in this study is 2, the number of nodes in the input layer is 28, the number of nodes in the hidden layer is 12, the number of nodes in the output layer is 4, the transfer function is the logsig + purelin function, and the training function is trainlr. We used the *dT* matrix to represent the 12-month supply chain performance evaluation results of automobile manufacturers’ output by the model in 2021.

\begin{eqnarray*}\cfsize[7.5pt][8pt]dT=1{0}^{-3} \left[ \begin{array}{@{}cccccccccccc@{}} \displaystyle 0.370&\displaystyle 0.003&\displaystyle 0.000&\displaystyle 0.000&\displaystyle -0.119&\displaystyle 0.000&\displaystyle 0.000&\displaystyle 0.002&\displaystyle 0.151&\displaystyle 0.000&\displaystyle 0.234&\displaystyle 0.000\\ \displaystyle 0.200&\displaystyle 0.000&\displaystyle 0.001&\displaystyle 0.000&\displaystyle 0.600&\displaystyle 0.000&\displaystyle 0.000&\displaystyle 0.000&\displaystyle -0.223&\displaystyle 0.000&\displaystyle 0.037&\displaystyle 0.000\\ \displaystyle 0.204&\displaystyle -0.001&\displaystyle 0.000&\displaystyle 0.000&\displaystyle -0.039&\displaystyle 0.000&\displaystyle 0.001&\displaystyle 0.000&\displaystyle 3.131&\displaystyle 0.000&\displaystyle 0.337&\displaystyle 0.000\\ \displaystyle -0.130&\displaystyle 0.000&\displaystyle 0.000&\displaystyle 0.000&\displaystyle 0.278&\displaystyle 0.000&\displaystyle 0.000&\displaystyle 0.000&\displaystyle 0.011&\displaystyle 0.000&\displaystyle -0.133&\displaystyle 0.000 \end{array} \right] .\resetfsize \end{eqnarray*}

It can be seen from the *dT* results that the maximum error level is lower than 0.4%, which is an acceptable level. Therefore, S enterprise can obtain more accurate evaluation results using the supply chain performance evaluation index system and LMBP algorithm. The model is accurate and efficient.

In the above analysis process, we can see that the months when the supply chain performance of the automobile manufacturing enterprise is evaluated as “poor” are February and March, respectively. The 4-dimensional data is expressed as (1 0 0 0), the months when the supply chain performance is evaluated as “excellent” are November and December, and the 4-dimensional data is expressed as (0 0 0 1). We selected February as the object of decision optimization to discuss improving the supply chain performance optimization path in February.

The optimization model provides strategic guidance to optimize supply chain management and improve supply chain performance. For example, if you try to improve the performance by 75%, the normalized value of the customer order X8 should be increased from 0.664 to 0.69. It can be calculated from the reverse direction; the customer order should be increased from 26,300 in February to 32,800, while the material completeness rate X5 should be increased from 95% to 98.23%. As a result, by increasing the customer order and material integrity rate, the automotive manufacturing company can strengthen supply chain management and boost supply chain performance in the subsequent work.

## Conclusions

As enterprises face the challenge of increased product diversity, they are also encountering heightened competition and more diverse customer needs in upstream and downstream industrial markets. This raises the bar for the operational decision-making abilities of companies. In this study, we selected automobile manufacturers in China’s listed manufacturing industry as research samples. By combining industry-specific characteristics with the Delphi method, we identified 28 types of market status data indicators with significant impact on upstream and downstream industries. We were able to analyze and mine the data more effectively using data reduction techniques based on adaptive statistics. We pinpointed a highly critical feature subset of 10 indicators that can effectively reveal the state of the market and support efficient operational decision-making based on supply chain management. Using the LMBP algorithm, we developed a novel method for supply chain performance evaluation, offering strategic guidance for optimizing supply chain management and improving supply chain performance. Our study indicates that automobile manufacturing enterprises can enhance their supply chain management capability by improving customer order and material readiness rates.

However, we acknowledge some limitations in this study and suggest areas for future research and improvement in management practices, including:

(1) Considering the internal interaction mechanism in the significant data capability structure dimension research, there are still hierarchical distinctions within big data capabilities.

(2) Exploring the impact of industry differences on extensive data research, as different industries have different priorities in the application process of big data.

##  Supplemental Information

10.7717/peerj-cs.1369/supp-1 Supplemental Information 1 Sample datasets for practice

10.7717/peerj-cs.1369/supp-2 Supplemental Information 2 Code

## Additional Information and Declarations

### Data Availability

The following information was supplied regarding data availability: The data and code files are available in the Supplementary Files.

## References

[ref-1] Belekoukias I, Garza-Reyes JA, Kumar V (2014). The impact of lean methods and tools on the operational performance of manufacturing organizations. International Journal of Production Research.

[ref-2] Belton I, MacDonald A, Wright G, Hamlin I (2019). Improving the practical application of the Delphi method in group-based judgment: a six-step prescription for a well-founded and defensible process. Technological Forecasting and Social Change.

[ref-3] Bian W, Shang J, Zhang J (2016). Two-way information sharing under supply chain competition. International Journal of Production Economics.

[ref-4] Centobelli P, Cerchione R, Esposito E (2018). Aligning enterprise knowledge and knowledge management systems to improve efficiency and effectiveness performance: a three-dimensional Fuzzy-based decision support system. Expert Systems with Applications.

[ref-5] Chowdhury MMH, Quaddus M (2017). Supply chain resilience: conceptualization and scale development using dynamic capability theory. International Journal of Production Economics.

[ref-6] Craighead CW, Ketchen Jr DJ, Darby JL (2020). Pandemics and supply chain management research: toward a theoretical toolbox. Decision Sciences.

[ref-7] Deng W, Feng L, Zhao X, Lou Y (2020). Effects of supply chain competition on firms’ product sustainability strategy. Journal of Cleaner Production.

[ref-8] Fan S, Lau RYK, Zhao JL (2015). Demystifying big data analytics for business intelligence through the lens of marketing mix. Big Data Research.

[ref-9] Flynn BB, Koufteros X, Lu G (2016). On theory in supply chain uncertainty and its implications for supply chain integration. Journal of Supply Chain Management.

[ref-10] Gardi B, Hamza PA, Sabir BY, Aziz HM, Sorguli S, Abdullah NN, Al-Kake F (2021). Investigating the effects of Financial Accounting Reports on Managerial Decision Making in Small and Medium-sized Enterprises. Turkish Journal of Computer and Mathematics Education (TURCOMAT).

[ref-11] Goodarzian F, Taleizadeh AA, Ghasemi P, Abraham A (2021). An integrated sustainable medical supply chain network during COVID-19. Engineering Applications of Artificial Intelligence.

[ref-12] Kaur H, Singh SP, Garza-Reyes JA, Mishra N (2020). Sustainable stochastic production and procurement problem for resilient supply chain. Computers & Industrial Engineering.

[ref-13] Kiseľáková D, Šofranková B, Čabinová V, Šoltésová J (2018). Analysis of enterprise performance and competitiveness to streamline managerial decisions. Polish Journal of Management Studies.

[ref-14] Kościelniak H, Puto A (2015). BIG DATA in decision making processes of enterprises. Procedia Computer Science.

[ref-15] Li X, Li Y (2016). Chain-to-chain competition on product sustainability. Journal of Cleaner Production.

[ref-16] Lima-Junior FR, Carpinetti LCR (2020). An adaptive network-based fuzzy inference system to supply chain performance evaluation based on SCOR^®^ metrics. Computers & Industrial Engineering.

[ref-17] Mishra D, Gunasekaran A, Papadopoulos T, Childe SJ (2018). Big Data and supply chain management: a review and bibliometric analysis. Annals of Operations Research.

[ref-18] Nasution MI, Fahmi M, Prayogi MA (2020). The quality of small and medium enterprises performance using the structural equation model-part least square (SEM-PLS). Journal of Physics: Conference Series. IOP Publishing.

[ref-19] Nhemachena C, Murimbika ME (2018). Motivations of sustainable entrepreneurship and their impact of enterprise performance in Gauteng Province, South Africa. Business Strategy & Development.

[ref-20] Ouedraogo CA, Montarnal A, Gourc D (2022). Multimodal container ttransportation ttraceability and supply chain risk management: a review of methods and solutions. International Journal of Supply and Operations Management.

[ref-21] Perfilieva IG, Yarushkina NG, Afanasieva TV, Romanov AA (2016). Web-based system for enterprise performance analysis on the basis of time series data mining. Proceedings of the first international scientific conference intelligent information technologies for industry (IITI’16) Volume 1.

[ref-22] Qu Q, Liu C, Bao X (2021). E-commerce enterprise supply chain financing risk assessment based on linked data mining and edge computing. Mobile Information Systems.

[ref-23] Reda M, Kanga DB, Fatima T, Azouazi M (2020). Blockchain in health supply chain management: state of art challenges and opportunities. Procedia Computer Science.

[ref-24] Rompho N (2018). Operational performance measures for startups. Measuring Business Excellence.

[ref-25] Rudskoi AI, Baurova NI (2019). Technological heredity during the production and operation of structural materials. Russian Metallurgy.

[ref-26] Sang B (2021). Application of genetic algorithm and BP neural network in supply chain finance under information sharing. Journal of Computational and Applied Mathematics.

[ref-27] Schmidt CG, Wagner SM (2019). Blockchain and supply chain relations: a transaction cost theory perspective. Journal of Purchasing and Supply Management.

[ref-28] Tavasszy LA (2018). Innovation and technology in multimodal supply chains.

[ref-29] Tiwari S, Wee HM, Daryanto Y (2018). Big data analytics in supply chain management between 2010 and 2016: insights to industries. Computers & Industrial Engineering.

[ref-30] Truong HQ, Sameiro M, Fernandes AC, Sampaio P, Duong BAT, Duong HH, Vilhenac E (2017). Supply chain management practices and firms’ operational performance. International Journal of Quality & Reliability Management.

[ref-31] Wang J, Xu C, Zhang J, Zhong R (2022). Big data analytics for intelligent manufacturing systems: a review. Journal of Manufacturing Systems.

[ref-32] Wang C, Wang W, Huang R (2017). Supply chain enterprise operations and government carbon tax decisions considering carbon emissions. Journal of Cleaner Production.

[ref-33] Yan F, Zhang Q, Ye S, Ren B (2019). A novel hybrid approach for landslide susceptibility mapping integrating analytical hierarchy process and normalized frequency ratio methods with the cloud model. Geomorphology.

[ref-34] Wang Y, Geng X, Zhang F, Ruan J (2018). An immune genetic algorithm for multi-echelon inventory cost control of IOT based supply chains. IEEE Access.

[ref-35] Wu Y, Lu R, Yang J, Xu F (2021). Low-carbon decision-making model of online shopping supply chain considering the O2O model. Journal of Retailing and Consumer Services.

[ref-36] Zhang A, Wang JX, Farooque M, Wang Y, Choi T-M (2021). Multidimensional circular supply chain management: a comparative review of the state-of-the-art practices and research. Transportation Research Part E: Logistics and Transportation Review.

