# Supplementary figures and images for: The impact of supply chain management on a company’s operation and decision based on the multidimensional data analysis of upstream and downstream industry market states
Source: PeerJ Comput Sci. 2023 Jun 13;9:e1369. doi: 10.7717/peerj-cs.1369 (PMC10280593; doi:10.7717/peerj-cs.1369)

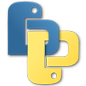

Supplement: Supplemental Information 2 [file peerj-cs-09-1369-s002.zip › ┤·┬δ/data/bpython.png]

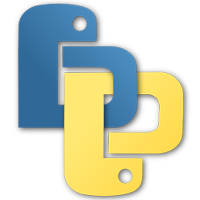

Supplement: Supplemental Information 2 [file peerj-cs-09-1369-s002.zip › ┤·┬δ/doc/sphinx/source/logo.png]
